# Genome-Wide Cell-Free DNA Test for Fetal Chromosomal Abnormalities and Variants: Unrestricted Versus Restricted Reporting

**DOI:** 10.3390/diagnostics12102439

**Published:** 2022-10-09

**Authors:** Angel H. W. Kwan, Xiaofan Zhu, Maria Mar Gil, Yvonne K. Y. Kwok, Isabella Y. M. Wah, Annie S. Y. Hui, Yuen-Ha Ting, Kwok-Ming Law, Doris Lau, Shuwen Xue, Kwong-Wai Choy, Daljit Sahota, Tak-Yeung Leung, Liona C. Poon

**Affiliations:** 1 Department of Obstetrics and Gynaecology, The Chinese University of Hong Kong, Hong Kong, China; 2 Genetics and Prenatal Diagnosis Center, Department of Obstetrics and Gynaecology, The First Affiliated Hospital of Zhengzhou University, Zhengzhou 450052, China; 3 Obstetrics and Gynecology Department, Hospital Universitario de Torrejón Torrejón de Ardoz, 28850 Madrid, Spain; 4 School of Medicine, Universidad Francisco de Vitoria (UFV), Pozuelo de Alarcón, 28223 Madrid, Spain; 5 Xcelom Ltd., Hong Kong, China

**Keywords:** genome-wide, cell-free DNA, screening for trisomies, down syndrome, additional findings, screening performance

## Abstract

This study aimed to compare the screening performance of genome-wide cfDNA testing for chromosomal abnormalities between two periods where additional findings were reported and not reported. Data were obtained from consecutive pregnant women with a singleton pregnancy at ≥10 weeks who requested cfDNA testing during 2015–2019. The performance of screening of the cfDNA test was determined by calculating the concordance rate, detection rate, and false-positive rate. Data from 3981 women were included. The no-result rates were similar between the two reporting periods (2.04% vs. 2.08%). Concordance rates for trisomy 21 and 18 were 100% and 100%, respectively. There were two cases tested high risk for trisomy 13, with a concordance rate of 0%. In total, 12 cases were high risk for any sex chromosome aneuploidy with an overall concordance of 75%, and 15 cases tested high risk for any rare autosomal trisomy, with a 13.3% concordance rate. The detection rates for trisomy 21 and 18 were 100% and 100%, respectively. For any SCA, the detection rate was 90%. For the two reporting periods, the combined false-positive rates were 0.93% and 0.17%, which were significantly different (*p* = 0.002). Restricting the reporting of additional findings from genome-wide cfDNA analysis has reduced the false-positive rate but without a reduction in the no-result rate.

## 1. Introduction

Several externally blinded validation and implementation studies published over the past few years have shown that, through analysis of cell-free (cf) DNA in maternal blood, it is now possible to effectively detect a high proportion of fetuses affected by trisomies 21, 18, and 13 at a much lower false-positive rate than all other existing screening methods [1,2,3,4,5,6,7,8,9,10,11]. A meta-analysis of combined data reports that, for singleton pregnancies, the detection rate for the three major trisomies by analysis of cfDNA in maternal blood is 99.7%, 97.8%, and 99.0% for trisomies 21, 18, and 13, respectively, at a combined false-positive rate of 0.13% [1]. Over the years, conventional cfDNA testing has expanded to cover the entire genome to detect sex-chromosome aneuploidies (SCAs) and a selection of microdeletion syndromes. More recently, it has been made possible to detect rare autosomal trisomies (RATs), structural chromosomal anomalies, and specific disease-causing copy-number variations (CNVs) by genome-wide cfDNA analysis [12,13,14,15,16,17,18,19,20,21]. However, the clinical utility of screening for these conditions is still controversial.

In our center, when self-financed genome-wide cfDNA testing was launched, in addition to risk results on trisomies 21, 18, and 13, SCAs and seven microdeletion syndromes (22q11 deletion syndrome, 1p36 deletion syndrome, 2q33.1 deletion syndrome, Cri-du-Chat syndrome, Angelman syndrome, Prader–Willi syndrome, Langer–Giedion syndrome), additional findings of genome-wide chromosomal aberrations at a resolution of 3 Mb or above were also reported. These additional findings included RATs, structural chromosomal imbalances, multiple aneuploidies, and other CNVs. In 2018, we changed our policy, and additional findings were no longer reported. 

The objectives of the study are to first report the performance of screening of the genome-wide cfDNA testing for trisomies 21, 18, and 13, SCAs and seven microdeletion syndromes and, second, compare the performance of screening of the genome-wide cfDNA testing between the two reporting periods. 

## 2. Materials and Methods

The data of this retrospective study were collected from consecutive pregnant women with singleton pregnancy at or beyond 10 weeks’ gestation attending the Prince of Wales Hospital, Hong Kong SAR, who opted for self-financed genome-wide cfDNA testing in screening for chromosomal abnormalities and genomic imbalances (SafeT21 Express^TM^, Xcelom, Hong Kong SAR) between May 2015 and November 2019. The cfDNA analysis was performed at our genome-wide cfDNA screening service provider laboratory. The study was approved by the Joint Chinese University of Hong Kong and New Territories East Cluster Clinical Research Ethics Committee (CREC, Ref no.: 2020:473). Before the test, the gestational age of the pregnancy was confirmed by the measurement of fetal crown-rump length, and major fetal abnormalities were excluded by ultrasound scan.

Pre-test counseling was provided by obstetricians, and written informed consent was obtained from all women for the cfDNA analysis. All women received a leaflet upon arrival for the hospital visit, which provided information on trisomies 21, 18, and 13, SCAs, and seven microdeletion syndromes. At the beginning of the service, women were informed that the cfDNA test detects 99% of fetuses with trisomy 21, 97% of fetuses with trisomy 18, and 92% of fetuses with trisomy 13; the screening performance of the latter two has improved to 98–99% over time [1,11], the cfDNA test has uncertain screening performance for the SCAs, and the seven microdeletion syndromes. Women were informed that the results from the cfDNA test are available in 7–10 days, but in about 2–3% of cases, the test does not give a result. During 2015–2017, women were also informed of the potential reporting on additional findings and their implications, such as the need for extra testing, including invasive testing, results of unknown significance, or parental findings. The turnaround time between the two time periods remained the same. 

Maternal blood was obtained by standard venepuncture (10 mL, in Streck cfDNA BCT^TM^ tube, La Vista, Nebraska, USA) and sent via courier to the laboratory for cfDNA testing (SafeT21 Express^TM^, Xcelom, Hong Kong SAR) [21,22,23,24,25,26]. Subsequent procedures and molecular tests involving the genome-wide cfDNA screening, including cfDNA isolation, library construction, sequencing, and bioinformatics analyses, were performed at the clinical laboratory of the genome-wide cfDNA screening service provider. Following library construction and amplification, the samples were sequenced on the Nextseq500 (Illumina, San Diego, CA, USA) with a minimum of 20 million read pairs per sample. The scope of detection and reporting of the genome-wide cfDNA screening included risk assessment for trisomy 21, trisomy 18, trisomy 13, SCAs, and seven microdeletion syndromes. During 2015–2017, additional findings of genome-wide chromosomal aberrations at a resolution of 3 Mb or above, including RATs, structural chromosomal imbalances, multiple aneuploidies, and other CNVs, were also reported. From 2018, analysis of chromosomal regions other than those that are pre-specified was masked, and the laboratory no longer reported on additional findings.

All patients with positive genome-wide cfDNA screening results were offered follow-up prenatal diagnostic confirmatory procedures. Diagnostic cytogenic and cytogenomic tests included conventional cytogenetic studies, quantitative fluorescent polymerase chain reaction (QF-PCR), fluorescence in situ hybridization (FISH), karyotype, and self-financed chromosomal microarray analysis (CMA) as deemed appropriate for the cases. DNA was extracted directly from uncultured samples (amniotic fluid, chorionic villus sample, placental or skin tissue) for molecular investigations. The majority of diagnostic tests reported in this study were performed at the Prenatal Genetic Diagnosis Laboratory of the Department of Obstetrics and Gynaecology, The Chinese University of Hong Kong. QF-PCR utilizing short tandem repeat markers was performed on each prenatally acquired sample to detect maternal cell contamination and polyploidy. The CMA test utilized a well-established Fetal DNA Chip v2.0 (CGH + SNP, Agilent Technologies Inc., Santa Clara, CA, USA); this employs a custom panel for prenatal diagnosis targeted at the loci of 100 common microdeletion, microduplication conditions, and uniparental disomy of clinical relevance at high resolution while providing whole genome coverage with a backbone resolution of 100 kb (http://www.obg.cuhk.edu.hk/wp-content/uploads/Fetal-DNA-Chip-v2.0_Leaflet.pdf). In the case when there was a high-risk result for a RAT but the discordant prenatal diagnostic result, when it was possible, following delivery, we collected five placental biopsies of 1 cm for karyotype though it was not an established protocol.

### 2.1. Pregnancy Outcome

Results of the cfDNA testing were recorded in a secure database. Results from invasive testing, obtained from laboratories, and pregnancy outcomes, obtained from obstetricians, general practitioners, or the patients by a telephone interview at least one month after delivery, were recorded in the same database. The outcomes were divided into firstly, trisomy 21, trisomy 18, trisomy 13, one of the SCAs, or one of the seven microdeletion syndromes if the QF-PCR, FISH, karyotype, or CMA of chorionic villi, amniotic fluid or neonatal blood demonstrated the relevant chromosomal abnormality or genomic aberration; secondly, no trisomy 21, trisomy 18, trisomy 13, SCAs, or seven microdeletion syndromes if the QF-PCR, FISH, karyotype or CMA of chorionic villi, amniotic fluid or neonatal blood was normal or the neonate did not phenotypically appear to have one of the predefined chromosomal abnormalities or genomic aberrations, thirdly, unknown QF-PCR, FISH, karyotype or CMA results because the pregnancies resulted in miscarriage or stillbirth and no diagnostic testing of fetal tissue was carried out, and fourthly, outcome unknown because the pregnancies were lost to follow up. Amongst those with outcomes, 13 of 58 women with high-risk cfDNA screening results have previously been included in a multicenter study to evaluate the concordance of cfDNA and CMA results [27].

### 2.2. Statistical Analysis

Descriptive data were presented in median and interquartile range (IQR) for continuous variables and in counts and percentages for categorical variables. Comparisons between outcome groups were by Mann–Whitney U-test for continuous variables and χ^2^-test or Fisher’s exact test for categorical variables.

The performance of screening for trisomy 21, trisomy 18, trisomy 13, SCAs, and the seven microdeletion syndromes by the genome-wide cfDNA test was determined by calculating the detection rate with 95% confidence intervals (CI). The confirmation rate of SCAs and the seven microdeletion syndromes by the genome-wide cfDNA test was determined with 95% CI. The combined false-positive rate (95% CI) was determined as the total number of high-risk cfDNA results in cases with the normal outcome. The screening performance was compared between the two reporting periods (2015–2017 vs. 2018–2019). 

The statistical software package SPSS 22.0 (SPSS Inc., Chicago, IL, USA) was used for data analyses.

## 3. Results

### 3.1. Study Population

During the study period, 3981 women with a viable singleton pregnancy were requested by choice and underwent self-financed cfDNA as first-line screening for chromosomal abnormalities. The median (interquartile range, IQR) maternal age was 34 years (31–37), with 43.7% of women with age ≥35 years old. The median (IQR) gestational age for a blood draw was 13.3 weeks gestation (12.4–14.6). The medial (IQR) fetal fraction was 11.9% (9.1–15.0).

### 3.2. Concordance Rate between Cell-Free DNA Test Results and Outcomes (Table 1)

Among the study population with available pregnancy outcomes (n = 3735; 93.8%), 3677 and 58 cases tested low risk and high risk, respectively, for chromosomal abnormalities. In total, 53 out of 58 high-risk cases (91.4%) underwent prenatal diagnostic testing, leading to an invasive testing rate of 1.4%. There were five cases that were tested high risk but with no invasive testing performed. One case had elevated amounts of chromosome 16; the fetus miscarried at 16 weeks with placental karyotype confirming trisomy 16. One case showed reduced amounts of DNA from chromosomes 1 and 6 in a woman with a history of known multiple large fibroids. For this case, the woman was counseled that multiple chromosome aberrations could be related to multiple fibroids or large fibroids [28]. The baby was delivered at term and appeared phenotypically normal; however, the placenta was not tested. Three cases had high-risk results for SCA. One case had borderline 45,X, based on a marginal z-score with reference to the fetal fraction (near to -3 cutoff for 45,X). A morphology scan subsequently showed no significant abnormalities and normal external genitalia, and the patient declined invasive testing. The remaining two cases tested high risk for XXY, and the patients opted for postnatal testing, which confirmed the diagnosis. 

**Table 1:** Concordance rates between cfDNA test results and outcomes.

Total Study Population, n = 3735				
Cell-Free DNA Results	Total	Outcome	n	Concordance (95% CI)
Low risk	3677 (98.4%)	Normal Mos 45,X[16]/46,XX[8])	3676 1	100% (99.9–100.0)
High risk for trisomy 21	20 (0.54%)	Trisomy 21	20	100% (83.9–100.0)
High risk for trisomy 18	5 (0.13%)	Trisomy 18	5	100% (56.6–100.0)
High risk for trisomy 13	2 (0.05%)	Normal	2	0% (0–65.8)
High risk for any SCA	12 (0.32%)	Normal Any SCA	3 9	75% (46.8–91.1)
• “Borderline 45,X”	3	Normal	2	33.3% (6.2–79.2)
		Mos X[7]/46,XX[31]	1	
• 45,X	3	Normal	2	0% (0–56.2)
		Isodicentric Y	1	
• Suspected X/XY	1	Normal	0	0% (0–79.4)
• 47,XXY	5	47,XXY	4	100% (56.6–100.0)
• “Borderline 45,X”	3	Normal	2	33.3% (6.2–79.2)
		Mos X[7]/46,XY[25]	1	
High risk for any RAT	15 (0.40%)	Normal	13	13.3% (3.7–37.9)
		Abnormal	2	
• Increased amount of chr 3	1	Normal	1	0% (0–79.4)
• Increased amount of chr 7	6	Normal	5	16.7% (3.0–56.4)
		Mos T7; 47 XX, + 7[9]/46,XX[21]	1	
• Increased amount of chr 8	1	Normal	1	0% (0–79.4)
• Increased amount of chr 9	1	Normal	1	0% (0–79.4)
• Increased amount of chr 10	1	Normal	1	0% (0–79.4)
• Increased amount of chr 11	1	Normal	1	0% (0–79.4)
• Increased amount of chr 16	2	Normal	1	50% (9.5–90.6)
		Trisomy 16	1	
• Increased amount of chr 20	1	Normal	1	0% (0–79.4)
• Reduced amount of chr 16	1	Normal	1	0% (0–79.4)
• Multiple chromosomal aberrations	1 (0.03%)	Normal	1	0% (0–79.4)
Any microdeletion syndromes	3 (0.08%)	Normal	2	33.3% (6.2–79.2)
		Abnormal	1	
• Cri du Chat	1	Cri du Chat	1	100% (20.7–100.0)
• 1 Mb deletion in Chr 15q11.2	1	Normal	1	0% (0–79.4)
• 19 Mb deletion in Chr 7q21.3	1	Normal	1	0% (0–79.4)
**Reporting period 2015–2017, n = 1952**				
Low risk	1914 (98.1%)	Normal	1913	100% (99.8–100.0)
		Mos 45,X[16]/46,XX[8])	1	
High risk for trisomy 21	11 (0.56%)	Trisomy 21	11	100% (74.1–100.0)
High risk for trisomy 18	4 (0.20%)	Trisomy 18	4	100% (51.0–100.0)
High risk for trisomy 13	1 (0.05%)	Normal	1	0% (0–79.4)
High risk for any SCA	4 (0.20%)	Any SCA	3	75% (30.1–95.4)
• “Borderline 45,X”	1	Normal	1	0% (0–79.4)
• 47,XXY	3	47,XXY	3	100% (43.9–100.0)
High risk for Any RAT	15 (0.77%)	Normal	13	13.3% (3.7–37.9)
		Abnormal	2	
• Increased amount of chr 3	1	Normal	1	0% (0–79.4)
• Increased amount of chr 7	6	Normal	5	16.7% (3.0–56.4)
		Mos T7; 47 XX, + 7[9]/46,XX[21]	1	
• Increased amount of chr 8	1	Normal	1	0% (0–79.4)
• Increased amount of chr 9	1	Normal	1	0% (0–79.4)
• Increased amount of chr 10	1	Normal	1	0% (0–79.4)
• Increased amount of chr 11	1	Normal	1	0% (0–79.4)
• Increased amount of chr 16	2	Normal	1	50% (9.5–90.6)
		Trisomy 16	1	
• Increased amount of chr 20	1	Normal	1	0% (0–79.4)
• Reduced amount of chr 16	1	Normal	1	0% (0–79.4)
Multiple chromosomal aberrations	1 (0.05%)	Normal	1	0% (0–79.4)
Microdeletion syndromes	2 (0.10%)	Normal	1	0% (0–65.8)
• 1 Mb deletion in Chr 15q11.2 • 19 Mb deletion in Chr 7q21.3	1 1	Normal Normal	1 1	0% (0–79.4) 0% (0–79.4)
**Reporting period 2018–2019, n = 1783**				
Low risk	1763 (98.9%)	Normal	1763	100% (99.8–100.0)
High risk for trisomy 21	9 (0.50%)	Trisomy 21	9	100% (70.1–100.0)
High risk for trisomy 18	1 (0.06%)	Trisomy 18	1	100% (20.7–100.0)
High risk for trisomy 13	1 (0.06%)	Normal	1	0% (0–79.4)
High risk for any SCA	8 (0.45%)	Normal	2	75.0% (40.9–92.9)
		SCA	6	
• Borderline 45,X	2	Normal	1	50% (9.5–90.6)
		Mos X[7]/46,XX[31]	1	
• 45,X	3	Normal	2	0% (0–56.2)
		Isodicentric Y	1	
• Suspected X/XY	1	Normal	0	0% (0–79.4)
• 47,XXY	2	47,XXY	1	100% (56.6–100.0)
		Mos X[7]/46,XY[25]	1	50% (9.5–90.6)
Microdeletion syndromes	1 (0.06%)	Normal	1	100% (0–20.7)
• Cri du Chat	1	Cri du Chat	1	100% (0–20.7)

SCA = sex chromosome aneuploidies; RAT = rare autosomal trisomies.

All cases (n = 27) with high-risk results for trisomy 21, trisomy 18, and trisomy 13 had invasive diagnostic testing. All high-risk cases of trisomy 21 (n = 20) and trisomy 18 (n = 5) were confirmed, giving 100% concordance. There were two cases that tested high risk for trisomy 13, with a concordance rate of 0%.

There were 12 cases that tested high risk for any SCA, with an overall concordance rate of 75%. Of note, a reported finding of borderline 45,X had 33.3% (one out of three) concordance, 45,X had 0% (zero out of three) concordance, while SCAs involving the Y-chromosome had 83.3% (five out of six) concordance with outcomes. Three cases tested high risk for any microdeletion syndromes, and the concordance rate was 33.3%, which was attributed to one case of Cri du Chat syndrome. All three cases had amniocentesis followed by karyotyping and CMA. 

Between the two reporting periods, the concordance rates for low-risk results and high-risk results for trisomy 21, 18, and 13 were the same. For any SCA, the overall concordance rates were 75% during both reporting periods. 

There were 15 cases that tested high risk for any RAT (Table 2), with a 13.3% concordance rate that was attributed to one case each tested high risk for an increased amount of chromosome 7 and chromosome 16. In the remaining 13 cases, we tested the placenta in eight cases, which showed confined placental mosaicism (CPM) in three (37.5%; one each of trisomy 16, trisomy 8, trisomy 20). For the case of CPM trisomy 16, the pregnancy was complicated by fetal growth restriction and preeclampsia, and the baby was delivered at 34 weeks by emergency Cesarean delivery with a birth weight of 1360 g. In the case of CPM trisomy 8, the patient developed preeclampsia at 37 weeks, and the baby was born by vaginal delivery following induction of labor with a birth weight of 2450 g. For the case with CPM trisomy 20, the patient had an uncomplicated pregnancy and delivered her baby at 39 weeks vaginally with a birth weight of 2870 g. For the five cases without evidence of CPM, all patients had an uncomplicated pregnancy. In the remaining five cases, we did not test the placenta, and amongst these cases, we could not rule out the possibility of CPM. Amongst these five cases, one case had preeclampsia at term (high risk for increased amount of chromosome 10), one case had superimposed preeclampsia on renal hypertension at term (high risk for increased amount of chromosome 9), and three cases had an uncomplicated pregnancy. Overall, 4 out of 15 (26.7%) cases testing high risk for any RAT were complicated by placental disorders. 

**Table 2:** Details of rare autosomal trisomy cases.

RAT	Diagnostic Testing	Testing Results	Pregnancy Outcome	Delivery at Term	Complications	Placenta
Increased amount of chr 3	Amniocentesis	46,XY	Live birth	Y	None	46,XY
Increased amount of chr 7	Amniocentesis	47XX, + 7[9]/46,XX [21]	MTOP	N	None	Not tested
	Amniocentesis	46,XY	Live birth		None	Not tested
	Amniocentesis	46,XX	Live birth	Y	None	46,XX
	Amniocentesis	46,XY	Live birth	Y	None	46,XY
	Amniocentesis	46,XY	Live birth		None	Not tested
	Amniocentesis	46,XX	Live birth	Y	None	46,XX
Increased amount of chr 8	Amniocentesis	46,XY	Live birth	Y	Preeclampsia	47,XY, + 8[4]/46,XY[46] (CPM)
Increased amount of chr 9	Amniocentesis	46XX	Live birth	Y	Superimposed preeclampsia on chronic renal hypertension	Not tested
Increased amount of chr 10	Amniocentesis	46,XX	Live birth	Y	Preeclampsia	Not tested
Increased amount of chr 11	Amniocentesis	46,XY	Live birth	Y	None	Not tested
Increased amount of chr 16	Amniocentesis	46,XX	Live birth	N	Fetal growth restriction	47,XX, + 16 (CPM)
	Not conducted		Miscarriage	N		47,XX, + 16
Increased amount of chr 20	Amniocentesis	46,XX	Live birth	Y	None	47,XX, + 20[2]/46,XX[28] (CPM)
Reduced amount of chr 16	Amniocentesis	Normal	Live birth	Y	None	46,XY

### 3.3. Test Performance of Cell-Free DNA Analysis (Table 3)

**Figure 1:**
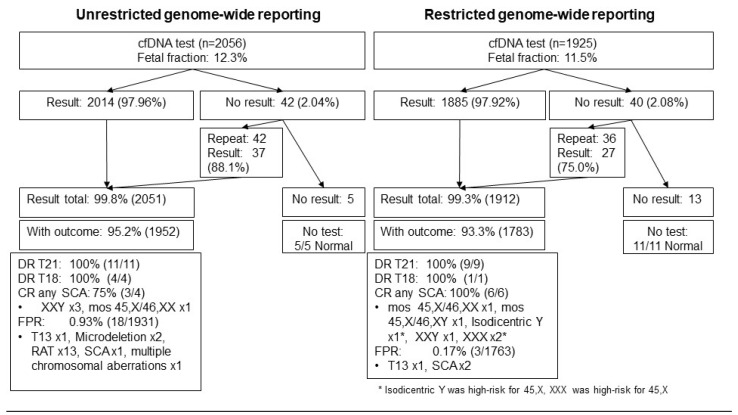
Overview of cfDNA and outcomes during the two reporting periods.

On the basis of the results of prenatal diagnostic testing, clinical examination of the neonates postnatally, or postnatal genetic confirmation, 20 cases of trisomy 21, five of trisomy 18, 10 of SCAs, and 1 case each of mosaic trisomy 7, trisomy 16, and Cri du Chat syndrome were detected (Figure 1).

**Table 3:** Screening performance of cfDNA results and pregnancy outcomes.

Total Study Population, n = 3735				
Outcome Group	Total	cfDNA Results	N, % (95% CI)	Pregnancy Outcomes, n (%)
Normal	3697 (99.0%)	Low risk	3676, 99.4 (99.1–99.6)	Live birth, 3644 (98.5) Miscarriage, 24 (0.7) Termination of pregnancy, 18 (0.5) Stillbirth, 12 (0.3)
		High risk	21, 0.57 (0.37–0.87)	
		• Trisomy 13	2, 0.05 (0.01–0.20)	
		• SCA	3, 0.08 (0.03–0.24)	
		• RAT	13, 0.35 (0.21–0.60)	
		• Multiple chromosomal aberrations	1, 0.03 (0.00–0.15)	
		• Microdeletion syndrome	2, 0.05 (0.01–0.20)	
Trisomy 21	20 (0.54%)	High risk for trisomy 21	20, 100 (83.9–100)	Live birth, 1 (5.0) Termination of pregnancy, 19 (95.0)
Trisomy 18	5 (0.13%)	High risk for trisomy 18	5, 100 (56.6–100)	Termination of pregnancy, 5 (100)
SCA	10 (0.27%)	Low risk	1, 10.0 (1.8–40.4)	Live birth, 7 (70.0) Termination of pregnancy, 3 (30.0)
		High risk for any SCA	9, 90.0 (59.6–98.2)	
• Mosaic 45,X/46,XX	2 (0.05%)	Low risk	1, 50.0 (9.5–90.6)	Termination of pregnancy, 2 (100)
		High risk for “borderline 45,X”	1, 50.0 (9.5–90.6)	
• Mosaic 45,X/46,XY	1 (0.03%)	High risk for XXY or low level of Y	1, 100 (20.7–100)	Live birth, 1 (100)
• Isodicentric Y	1 (0.03%)	High risk for 45,X	0, 0 (0–79.4)	Live birth, 1 (100)
• 47,XXX	2 (0.05%)	High risk for 45,X	0, 0 (0–65.8)	Live birth, 2 (100)
• 47,XXY	4 (0.11%)	High risk for 47,XXY	4, 100 (51.0–100)	Live birth, 4 (100)
Mos T7; 47 XX, + 7 [9]/46,XX [21]	1 (0.03%)	High risk for increased chr 7	1, 100 (20.7–100)	Termination of pregnancy, 1 (100)
Trisomy 16	1 (0.03%)	High risk for increased chr 16	1, 100 (20.7–100)	Miscarriage, 1 (100)
Cri du Chat	1 (0.03%)	High risk for Cri du Chat	1, 100 (20.7–100)	Termination of pregnancy, 1 (100)
**Reporting period 2015–2017, n = 1952**				
Normal	1931 (99.0%)	Low risk	1913, 99.0 (98.5–99.4)	Live birth, 1898 (98.2) Miscarriage, 12 (0.6) Termination of pregnancy, 14 (0.7) Stillbirth, 8 (0.4)
		High risk	18, 0.93 (0.59–1.47)	
		• Trisomy 13	1, 0.05 (0.01–0.30)	
		• RAT	13, 0.67 (0.39–1.15)	
		• SCA	1, 0.05 (0.01–0.30)	
		• Multiple chromosomal aberrations	1, 0.10 (0.01–0.29)	
		• Microdeletion syndrome	2, 0.10 (0.03–0.38)	
Trisomy 21	11 (0.56%)	High risk for trisomy 21	11, 100 (74.1–100)	Live birth, 1 (9.1) Termination of pregnancy, 10 (90.9)
Trisomy 18	4 (0.20%)	High risk for trisomy 18	4, 100 (51.0–100)	Termination of pregnancy, 4 (100)
SCA	4 (0.20%)	Low risk	1, 25.0 (4.6–69.9)	Live birth, 3 (75.0) Termination of pregnancy, 1 (25.0)
		High risk for any SCA	3, 75.0 (30.1–95.4)	
• Mosaic 45,X/46,XX	1 (0.05%)	Low risk	0, 0 0, 0 (0–79.4)	Termination of pregnancy, 1 (100)
• 47,XXY	3 (0.15%)	High risk for 47,XXY	3, 100 (43.9–100)	Live birth, 3 (100)
Mos T7; 47 XX, + 7 [9]/46,XX [21]	1 (0.05%)	High risk for increased chr 7	1, 100 (20.7–100)	Termination of pregnancy, 1 (100)
Trisomy 16	1 (0.03%)	High risk for increased chr 16	1, 100 (20.7–100)	Miscarriage, 1 (100)
**Reporting period 2018–2019, n = 1783**				
Normal	1766 (99.1%)	Low risk	1763, 99.8 (99.5–100)	Live birth, 1746 (98.9) Miscarriage, 12 (0.7) Termination of pregnancy, 4 (0.2) Stillbirth, 4 (0.2)
		High risk	3, 0.17 (0.06–0.50)	
		• Trisomy 13	1, 0.06 (0.01–0.33)	
		• SCA	2, 0.11 (0.03–0.41)	
Trisomy 21	9 (0.50%)	High risk for trisomy 21	9, 100 (70.1–100)	Termination of pregnancy, 9 (100)
Trisomy 18	1 (0.06%)	High risk for trisomy 18	1, 100 (20.7–100)	Termination of pregnancy, 1 (100)
SCA	6 (0.34%)	High risk for any SCA	6, 100 (61.0–100)	Live birth, 4 (66.7) Termination of pregnancy, 2 (33.3)
• Mosaic 45,X/46,XX	1 (0.06%)	High risk for borderline 45,X	1, 100 (20.7–100)	Termination of pregnancy, 1 (100)
• Mosaic 45,X/46,XY	1 (0.06%)	High risk for XXY or low level of Y	1, 100 (20.7–100)	Live birth, 1 (100)
• Isodicentric Y	1 (0.06%)	High risk for 45,X	0, 0 (0–79.4)	Live birth, 1 (100)
• 47,XXX	2 (0.11%)	High risk for 45,X	0, 0 (0–65.8)	Live birth, 1 (100)
• 47,XXY	1 (0.06%)	High risk for 47,XXY	1, 100 (20.7–100)	Live birth, 1 (100)
Cri du Chat	1 (0.06%)	Cri du Chat	1, 100 (20.7–100)	Termination of pregnancy, 1 (100)

A total of 21 cases had false-positive results, which had normal pregnancy outcomes. cfDNA analysis detected a high risk for two cases of trisomy 13, three cases for any SCA, thirteen cases of RAT, one case of multiple chromosomal aberrations, and two cases of microdeletion syndromes. The combined false-positive rate was 0.57% (0.37–0.87). The detection rates for trisomy 21 and trisomy 18 were 100% (95% CI 83.9–100) and 100% (95% CI 56.6–100), respectively. The confirmation rate for any SCA was 75% (95% CI 46.8–91.1). A low level of sex chromosome mosaicism may have contributed to the discordant result for the SCA. Specifically, for 47,XXY, the confirmation rate was 100% (95% CI 51.0–100); one of two cases of mosaic 45,X/46,XX tested high -risk for borderline 45,X, one case of mosaic 45,X/46,XY tested high risk for the low level of Y, one case of isodicentric Y tested high risk for 45,X and two cases of XXX tested high risk for 45,X. For the cases of mosaic trisomy 7, trisomy 16, and Cri du Chat syndrome, they were detected by cfDNA testing. By restricting the reporting of additional findings from genome-wide cfDNA analysis, the combined false-positive rate reduced from 0.93% (95% CI 0.59–1.47) to 0.17% (95% CI 0.06–0.50; *p* = 0.002). The detection rates for trisomy 21 and 18 between the two reporting periods were the same, whilst the confirmation rates for any SCA were 75% and 100%, respectively. However, the cases of isodicentric Y and XXX during the second study period were detected by the high-risk results of 45,X.

Regarding pregnancy outcomes, 19/20 (95.0%), 3/3 (100%), and 2/10 (20.0%) women with fetal trisomy 21, trisomy 18, and any SCA underwent termination of pregnancy. The two SCA cases that opted for termination were two cases of mosaic 45,X. One of the mosaic 45,X cases had ultrasound findings of type B interrupted aortic arch, ventricular septal defect, and aortic stenosis, whilst the other case did not have ultrasound abnormality. Cases were genetically confirmed. For the cases with fetal mosaic trisomy 7 and Cri du Chat syndrome, both women opted for a termination of pregnancy. For the cases with no result, all had normal pregnancy outcomes.

### 3.4. Frequency of No Result

There were 82 (2.06%) cases with no result from the first blood draw, including 18 (0.45%) as a result of the pre-laboratory error, 21 (0.53%) because of interference, three results (0.08%) were equivocal, and 38 (0.95%) because of low fetal fraction (Table 4). In total, 78 cases had a second blood draw, and 64 (82.1%) obtained results, of which there was one case each that tested high risk for trisomy 21 and trisomy 18, two cases tested high risk for SCAs, and one case tested high risk for RAT. Three cases had a third blood draw, one case had no result because of low fetal fraction, one case tested high risk for SCA, and one case was low risk. During the reporting period of 2015–2017, the no-result rate was 2.04% (42/2057), and subsequent blood draw led to available results in 37/42 (88.1%). Similarly, during the reporting period of 2018–2019, the no-result rate was 2.08% (40/1925), and subsequent blood draw led to available results in 27/36 (75.0%). The no-result rates were not significantly different between the two reporting periods (*p* > 0.999).

**Table 4:** Reasons for no result.

Total Study Population, n = 82/3981 (2.06%)					
First Draw	Total (n = 82)	Second Draw	n	Third Draw	n
Pre-laboratory error	18 (0.45%)	Low risk High risk for trisomy 18 Pre-laboratory error No redraw	15 1 1 1		
Interference	21 (0.53%)	Low risk Interference No redraw	12 8 1	High risk for SCA	1
Equivocal	3 (0.08%)	Low risk High risk for SCA High risk for RAT	1 1 1		
Low fetal fraction	38 (0.95%)	Low risk Low fetal fraction High risk for trisomy 21 High risk for SCA No redraw	27 7 1 1 2	Low risk/low fetal fraction	1/1
Others	2 (0.05%)	Low risk	2		
**Reporting period 2015–2017, n = 42/2056 (2.04%)**					
Pre-laboratory error	8 (0.39%)	Low risk High risk for trisomy 18	7 1		
Interference	10 (0.49%)	Low risk Interference	9 1		
Equivocal	3 (0.15%)	Low risk High risk for SCA High risk for RAT	1 1 1		
Low fetal fraction	19 (0.92%)	Low risk Low fetal fraction High risk for trisomy 21 High risk for SCA	13 4 1 1	Low fetal fraction	1
Others	2 (0.09%)	Low risk	2		
**Reporting period 2018–2019, n = 40/1925 (2.08%)**					
Pre-laboratory error	10 (0.52%)	Low risk Pre-laboratory error No redraw	8 1 1		
Interference	11 (0.57%)	Low risk Interference No redraw	3 7 1	High risk for SCA	1
Low fetal fraction	19 (0.99%)	Low risk Low fetal fraction No redraw	14 3 2	Low risk	1

SCA = sex chromosome aneuploidies; RAT = rare autosomal trisomies.

## 4. Discussion

The main finding of this study is that by restricting the reporting of cfDNA results to the three major fetal trisomies, SCAs, and seven microdeletion syndromes and avoiding the reporting of additional findings such as RATs, structural chromosome imbalances, all CNVs or multiple aneuploidies, the false-positive rate can be significantly lowered from 0.93% (95% CI 0.59–1.47) to 0.17% (95% CI 0.06–0.50), but without a reduction in the no-result rate.

Our results on the cfDNA test performance are in agreement with previous studies demonstrating the better performance of the cfDNA test for the major trisomies than SCAs, especially when considering 45,X [1,12,14,29,30,31,32,33,34]. However, it has been difficult to assess the detection rates for SCAs and microdeletion syndromes when CMA has not been available for all cases, and outcome ascertainment has relied on neonatal examination. Unlike trisomies 21, 18, and 13, neonates with SCAs other than 45,X with prenatal ultrasound findings, as well as rare microdeletion syndromes, are often phenotypically normal. Consequently, it is likely that we have underestimated the true prevalence of these abnormalities in our population and therefore overestimated the potential sensitivity of prenatal cfDNA testing. In our study, we took the precaution of reporting the confirmation rate for SCAs and microdeletion syndromes rather than the detection rate. While there is general consensus on the value of testing for the three major trisomies and fetal sex, the clinical utility for screening for SCAs and other chromosomal abnormalities is still debatable.

In our clinical setting, the genome-wide cfDNA test is a commercially available one that tests for the three major fetal trisomies, SCAs, and seven microdeletion syndromes. Women are given the option to opt out of reporting SCAs and microdeletion syndromes, but the uptake for this option is low. At present, there are no prenatal screening programs for SCAs, mainly due to the significant variations in their clinical manifestations and the lack of an accurate method of screening and intervention that could improve outcomes. Therefore, the main prenatal diagnosis of SCAs currently depends on the detection of ultrasound features of the severe type of 45,X, and the role of cfDNA testing for this purpose deserves further research. Of note, no cases of structural chromosomal aberration were detected in our population. As compared to the TRIDENT study, with a study population of 73,239 women, the frequency of structural chromosomal aberration was 0.16%, with a positive predictive value of 32%; therefore, our population may be too small to detect such rare abnormalities by cfDNA testing [31].

We reported 15 cases with high-risk results for RATs (0.40%), but only two cases (13.3%) were confirmed in fetal tissue. We tested the placenta in 8 out of 13 cases, which showed CPM in three (37.5%). In the remaining five cases, we did not test the placenta, and amongst these cases, we could not rule out the possibility of CPM. Our reported concordance rate of the cfDNA testing for RATs is comparable to that of the Dutch TRIDENT-2 study that has recently reported a positive predictive value of 6% for RATs through the evaluation of the implementation of cfDNA testing as first-line screening [27]. Additional reporting of these pregnancies to be high risk for RATs does not appear to have an additional benefit because of the low concordance rate and uncertain detection rate. In cases of nonmosaic autosomal trisomies, most will have resulted in first-trimester miscarriage, which is expected to be evident by the first-trimester cfDNA testing. Besides creating additional maternal anxiety from a positive cfDNA result, this may also lead to a higher invasive prenatal testing rate and possibly termination rate. For the two RATs detected through cfDNA testing and confirmed via invasive testing, a case of mosaic trisomy 7 and trisomy 16 ended up in a termination of pregnancy and a miscarriage, respectively. RATs are the most frequently detected mosaic abnormalities in chorionic villi sampling, with an incidence rate of 54.5% [35]. The presence of self-correcting mechanisms in a mosaic embryo allows for a normal pregnancy with a healthy baby being born. Trisomy 16 is the most common trisomy reported in miscarriages and is estimated to occur in 1.5% of all pregnancies. Existing literature has reported complications associated with mosaic trisomy 16, which include fetal growth restriction, preterm birth, pre-eclampsia, and intrauterine fetal demise [36].

Whether these high-risk cases for RATs need further investigations when amniotic fluid samples test negative for RATs in order to identify CPM to prevent possible related adverse fetal and maternal outcomes by intensive prenatal monitoring remains controversial. In a recent study evaluating outcomes in pregnancies with CPM and implications for prenatal cfDNA screening, the authors concluded that with the exception of trisomy 16, the incidence of adverse outcomes related to a RAT confined to the placenta was very low, and therefore, prenatal genome-wide cfDNA screening for such conditions would be of minimal benefit [37]. However, population studies comparing restricted and unrestricted cfDNA testing to evaluate the utility of disclosing additional findings have concluded that such an approach is controversial [16,38]. Our data do not provide evidence to support screening for RATs. The additional information makes counseling difficult and may increase maternal anxiety. The genotype–phenotype correlation may be unknown due to the variability of the location of the abnormal fetal cell line. In our experience, only CPM trisomy 16 was associated with severe placental complications, while CPM trisomy 8 and possibly CPM trisomy 10 were associated with mild placental complications. However, it is unknown whether these pregnancy complications are more prevalent in the CPM population versus that of the general population; therefore, screening for pregnancy complications via cfDNA testing is still currently not supported by professional societies.

Similar to every screening method, when using cfDNA testing for screening of chromosomal abnormalities, there is a chance of failure to obtain a result. In our study, both unrestricted and restricted reporting demonstrated no-result rates of about 2%, which are comparable with the current literature [39,40]. The reasons for no result can largely be grouped into different categories: firstly, due to pre-laboratory errors where the blood failed quality control, for example, hemolysis or blood clotting; secondly, due to assay interference and thirdly, due to low fetal fraction, with the latter being the main reason for test failure. In our study, test failure resulting from low fetal fraction occurred in about 0.95% in both reporting periods. A meta-analysis by *Gil* et al. reported failure rates ranging from 0 to 12.2%, with no possible explanations for the wide range in failure rates between the studies [1]. Maternal age, weight, racial origin, parity, gestational age, method of conception, and serum levels of free β-human chorionic gonadotropin and pregnancy-associated plasma protein-A were found to be independent predictors of cfDNA test failure [1].

Additionally, trisomies 13 and 18 have been shown to be associated with a higher no-result rate in relation to lower fetal fractions, likely due to smaller placental mass [41,42]. As such, a pregnancy without cfDNA result because of the low fetal fraction can be considered at a higher risk of trisomy 13 or 18. Amongst our no-result cases, we had one case each that subsequently tested high risk for trisomy 21 (1.2%) and trisomy 18 (1.2%). It is therefore advisable to repeat the blood draw in order to ascertain a definitive cfDNA result. If concurrent first-trimester screening with ultrasound and serum biochemical markers has been performed, it would be beneficial to evaluate these results before deciding to proceed to invasive testing or a redraw. A detailed ultrasound scan for features of trisomies 13 and 18 would be useful for determining the need for further invasive testing [42]. It is reassuring to learn that in our study, those with no result had an uncomplicated pregnancy.

One of the main strengths of our study is that we were able to obtain 94% of pregnancy outcomes. For the cases that were tested high risk for a RAT but with negative results from prenatal diagnostic testing, we obtained placental biopsy in 62% of cases to look for CPM. In addition to genetic testing, maternal and fetal outcomes were both documented, which allowed for correlation with any positive cfDNA results. Our study is also the first to compare the screening performance between restricted versus unrestricted genome-wide cfDNA screening after the change in policy in 2018, therefore demonstrating the impact of reporting additional chromosomal aberrations on pregnancy outcomes and on the invasive testing rate.

The main limitation of our study relates to the outcome ascertainment, as discussed above. For this reason, conclusions on screening performance of the cfDNA testing for SCAs, microdeletion syndromes, and other additional findings must be interpreted with caution. Additionally, the number of cases affected by these conditions was very small for this purpose. However, in order to properly evaluate the clinical implications of prenatal screening for all these chromosomal or genetic abnormalities, it would be necessary to not only perform karyotyping and CMA analysis in the entire population but also follow up on the neonates throughout their lives in order to understand the disease, its implications, and the consequences of early detection. Another limitation is that we should have tested the same women by restrictive and unrestrictive cfDNA analysis in order to allow comparisons, especially of those related to the no-result rate between strategies. However, comparing two consecutive cohorts from the same geographical area in such a short time frame ensures that differences in population characteristics are minimal. Our small number of affected cases may also have contributed to the high concordance rates for trisomies 21 and 18.

One of the main benefits of incorporating cfDNA testing in pregnancy care is the significant reduction in the number of invasive tests and their associated risks. Using the first-trimester combined screening, the detection rates for the three major trisomies are about 90–95% at a 5% false positive rate [43]. In contrast, these rates improve to >99% and about 1% when incorporating cfDNA unrestricted screening in our study population. With the introduction of restricted screening, the false-positive rate further decreased to 0.17% without losing accuracy. Besides the reduction in procedure-related risks, this may also help reduce maternal anxiety caused as a result of a positive screening result or by the diagnosis of conditions of unknown significance. Therefore, it is important that before implementing screening of any condition, clinicians must balance the potential benefits against the potential harms, but at the same time, we also encourage researchers to continue developing or improving the test in order to help solve all these questions.

## 5. Conclusions

In conclusion, restricting the reporting of additional findings from genome-wide cfDNA analysis allows a reduction in the false-positive rate, but there is no reduction in the no-result rate.

## Data Availability

The data that support the findings of this study are available on request from the corresponding author. The data are not publicly available due to privacy or ethical restrictions.
